# Supplementary material for: ProteinShader: illustrative rendering of macromolecules
Source: BMC Struct Biol. 2009 Mar 30;9:19. doi: 10.1186/1472-6807-9-19 (PMC2672931; doi:10.1186/1472-6807-9-19)
Supplement: Additional file 1 — ProteinShader program without source code. This compressed file contains the complete ProteinShader program including associated libraries, but no source code. A README.txt file gives an overview of the ProteinShader distribution, and the index.html file in the help subdirectory has directions on getting started with the program as well as a set of tutorials. [file 1472-6807-9-19-S1.zip › ProteinShader-beta-0_9_4-binary/help/api/org/proteinshader/structure/visitor/SFWriterVisitor.html]

SFWriterVisitor (ProteinShader API)


|  |  |  |
| --- | --- | --- |
| **Overview** \| **Package** \| **Class** \| **Use** \| **Tree** \| **Deprecated** \| **Index** \| **Help** | |  |
| **PREV CLASS**   **NEXT CLASS** | **FRAMES**    **NO FRAMES**     **All Classes** |  |
| SUMMARY: NESTED \| FIELD \| CONSTR \| METHOD | DETAIL: FIELD \| CONSTR \| METHOD |  |


---


## org.proteinshader.structure.visitor Class SFWriterVisitor

```
java.lang.Object
  org.proteinshader.structure.visitor.Visitor
      org.proteinshader.structure.visitor.SFWriterVisitor
```

---

``` public class SFWriterVisitor extends Visitor ```

A simple file writer subclass of Visitor that writes to a file all of
the data from each Model in a Structure. This class is intended to be
useful for development and debugging.
The output is a simple object dump that announces each object, writes
its attributes, and then traverses the objects associations so that
they can be written.

---

| **Field Summary** | |
| --- | --- |
| `static int` | `CHAR_PER_LINE` |
| `static int` | `INDENTATION_FACTOR` |


**Constructor Summary**

`SFWriterVisitor()`
  
          Constructs a SFWriterVisitor.

`SFWriterVisitor(String outputFile)`
  
          Constructs a SFWriterVisitor that will write to the output file
given as an argument.


| **Method Summary** | |
| --- | --- |
| `void` | `closeOutputFile()`             This method flushes the output buffer to make sure that all data is written before closing the output file. |
| `void` | `flush()`             This method forces the writer to immediately write all data in its buffer. |
| `void` | `openOutputFile(String outputFile)`             Opens a buffered writer using the filename given as an argument. |
| `void` | `visit(AminoAcid aminoAcid)`             Writes all information held by an AminoAcid. |
| `void` | `visit(Atom atom)`             Writes all information held by an Atom. |
| `void` | `visit(BetaStrand betaStrand)`             Writes all information held by a BetaStrand. |
| `void` | `visit(Bond bond)`             Writes all information held by a Bond. |
| `void` | `visit(Chain chain)`             Writes all information held by a Chain. |
| `void` | `visit(Helix helix)`             Writes all information held by a Helix. |
| `void` | `visit(Heterogen heterogen)`             Writes all information held by a Heterogen. |
| `void` | `visit(Loop loop)`             Writes all information held by a Loop. |
| `void` | `visit(Model model)`             Writes all information held by a Model. |
| `void` | `visit(Segment segment)`             Writes information on a Segment. |
| `void` | `visit(Structure structure)`             Writes a Structure's data to a simple text file. |
| `void` | `visit(Water water)`             Writes all information held by a Water. |
| `void` | `writeDescription(Description description)`             writeDescription() - writes all information on a Description. |

**Methods inherited from class org.proteinshader.structure.visitor.Visitor**

`clear, includeAAHetAndWater, setDebug, setMode, setMode, setMode, setMode, setMode, setMode, setVisitSegments`

**Methods inherited from class java.lang.Object**

`clone, equals, finalize, getClass, hashCode, notify, notifyAll, toString, wait, wait, wait`

**Field Detail**

### CHAR\_PER\_LINE

```
public static final int CHAR_PER_LINE
```

**See Also:**: Constant Field Values

---


### INDENTATION\_FACTOR

```
public static final int INDENTATION_FACTOR
```

**See Also:**: Constant Field Values


**Constructor Detail**

### SFWriterVisitor

```
public SFWriterVisitor()
```

:   Constructs a SFWriterVisitor.

---


### SFWriterVisitor

```
public SFWriterVisitor(String outputFile)
                throws VisitorException
```

:   Constructs a SFWriterVisitor that will write to the output file
    given as an argument.


**Method Detail**

### openOutputFile

```
public void openOutputFile(String outputFile)
                    throws VisitorException
```

:   Opens a buffered writer using the filename given as an argument.

---


### flush

```
public void flush()
           throws VisitorException
```

:   This method forces the writer to immediately write all data in its
    buffer.

    :   **Throws:**: `VisitorException` - if an IO error occurs.

---


### closeOutputFile

```
public void closeOutputFile()
                     throws VisitorException
```

:   This method flushes the output buffer to make sure that all data
    is written before closing the output file.
    After calling closeOutputFile(), further visit() or flush()
    invocations will result in a VisitorException, unless
    openOutputFile() has been called to open up another output file
    to write to.

    :   **Throws:**: `VisitorException` - if an IO error occurs.

---


### visit

```
public void visit(Structure structure)
           throws VisitorException
```

:   Writes a Structure's data to a simple text file.

    :   **Overrides:**: `visit` in class `Visitor`
    :   **Parameters:**: `structure` - the Structure to write. **Throws:**: `VisitorException` - if an IO error occurs.

---


### visit

```
public void visit(Model model)
           throws VisitorException
```

:   Writes all information held by a Model.

    :   **Overrides:**: `visit` in class `Visitor`
    :   **Parameters:**: `model` - the Model to write. **Throws:**: `VisitorException` - if a problem occurs while writing.

---


### visit

```
public void visit(Chain chain)
           throws VisitorException
```

:   Writes all information held by a Chain.

    :   **Overrides:**: `visit` in class `Visitor`
    :   **Parameters:**: `chain` - the Chain to write. **Throws:**: `VisitorException` - if an IO error occurs.

---


### visit

```
public void visit(Loop loop)
           throws VisitorException
```

:   Writes all information held by a Loop.

    :   **Overrides:**: `visit` in class `Visitor`
    :   **Parameters:**: `loop` - the Loop to write. **Throws:**: `VisitorException` - if an IO error occurs.

---


### visit

```
public void visit(Helix helix)
           throws VisitorException
```

:   Writes all information held by a Helix.

    :   **Overrides:**: `visit` in class `Visitor`
    :   **Parameters:**: `helix` - the Helix to write. **Throws:**: `VisitorException` - if an IO error occurs.

---


### visit

```
public void visit(BetaStrand betaStrand)
           throws VisitorException
```

:   Writes all information held by a BetaStrand.

    :   **Overrides:**: `visit` in class `Visitor`
    :   **Parameters:**: `betaStrand` - the BetaStrand to write. **Throws:**: `VisitorException` - if an IO error occurs.

---


### visit

```
public void visit(Segment segment)
           throws VisitorException
```

:   Writes information on a Segment.

    :   **Overrides:**: `visit` in class `Visitor`
    :   **Parameters:**: `segment` - the Segment to write. **Throws:**: `VisitorException` - if an IO error occurs.

---


### visit

```
public void visit(AminoAcid aminoAcid)
           throws VisitorException
```

:   Writes all information held by an AminoAcid.

    :   **Overrides:**: `visit` in class `Visitor`
    :   **Parameters:**: `aminoAcid` - the AminoAcid to write. **Throws:**: `VisitorException` - if an IO error occurs.

---


### visit

```
public void visit(Heterogen heterogen)
           throws VisitorException
```

:   Writes all information held by a Heterogen.

    :   **Overrides:**: `visit` in class `Visitor`
    :   **Parameters:**: `heterogen` - the Heterogen to write. **Throws:**: `VisitorException` - if an IO error occurs.

---


### visit

```
public void visit(Water water)
           throws VisitorException
```

:   Writes all information held by a Water.

    :   **Overrides:**: `visit` in class `Visitor`
    :   **Parameters:**: `water` - the Water to write. **Throws:**: `VisitorException` - if an IO error occurs.

---


### visit

```
public void visit(Atom atom)
           throws VisitorException
```

:   Writes all information held by an Atom.

    :   **Overrides:**: `visit` in class `Visitor`
    :   **Parameters:**: `atom` - the Atom to write. **Throws:**: `VisitorException` - if an IO error occurs.

---


### visit

```
public void visit(Bond bond)
           throws VisitorException
```

:   Writes all information held by a Bond.

    :   **Overrides:**: `visit` in class `Visitor`
    :   **Parameters:**: `bond` - the Bond to write. **Throws:**: `VisitorException` - if an IO error occurs.

---


### writeDescription

```
public void writeDescription(Description description)
                      throws WriterVisitorException
```

:   writeDescription() - writes all information on a Description.

    :   **Parameters:**: `description` - the Description to write. **Throws:**: `WriterVisitorException` - if an IO error occurs.


---


|  |  |  |
| --- | --- | --- |
| **Overview** \| **Package** \| **Class** \| **Use** \| **Tree** \| **Deprecated** \| **Index** \| **Help** | |  |
| **PREV CLASS**   **NEXT CLASS** | **FRAMES**    **NO FRAMES**     **All Classes** |  |
| SUMMARY: NESTED \| FIELD \| CONSTR \| METHOD | DETAIL: FIELD \| CONSTR \| METHOD |  |


---

# *Copyright © 2007-2008*
